# Vascular endothelial growth factor-tyrosine kinase inhibitors: Novel mechanisms, predictors of hypertension and management strategies

**DOI:** 10.1016/j.ahjo.2022.100144

**Published:** 2022-05-26

**Authors:** Javier A. Gomez

**Affiliations:** John H Stroger Jr Hospital of Cook County, United States

**Keywords:** Hypertension, Cancer, Cardio-oncology, Tyrosine kinase inhibitors

## Abstract

Vascular endothelial growth factor inhibition has become a cornerstone of cancer therapy. Significant adverse effect is associated with these therapies, with hypertension among the most prevalent. We aim to provide a current review of the mechanisms by which these therapies induce hypertension, the risk factors and predictors of the development of hypertension, and management strategies to minimize the risk of cardiovascular complications in patients receiving vascular endothelial growth factor inhibition as part of cancer treatment.

The human vascular endothelial growth factor (VEGF) family is comprised of five related glycoproteins: VEGF-A, VEGF-B, VEGF-C, VEGF-D, and placental growth factor (PIGF), which interact with three receptor tyrosine kinases, the VEGF receptor (VEGFR) 1, VEGFR 2 and VEGFR 3. Upon ligand binding, various intracellular signals and mediators are activated through which the vascular endothelial growth factor signaling pathway (VSP) exerts its effects [1], [2]. The effects of VEGF are primarily driven by increases in levels of nitric oxide (NO) and prostacyclin 2 (PGI2). Increased NO and PGI2 production leads to enhanced vascular permeability, vasorelaxation, enhanced endothelial survival, and facilitate angiogenesis [3], [4].

Tumor angiogenesis is considered one of the hallmarks of cancer [5]. Neovasculature supplies the tumor with the necessary substrates for proliferation while removing waste products, making it a fundamental step for tumor growth, progression, and metastasis [6]. Based on these observations, targeting blood vessel proliferation was introduced to treat various malignancies. Several strategies to inhibit the effects of the VSP have been used and can be divided into two categories: specific agents blocking the VEGF/VEGFR actions or small molecule tyrosine kinase inhibitors (TKIs), which block several different parts of the VSP and produce a potent inhibition of the VSP effects. The monoclonal antibody bevacizumab was the initial drug developed, directly neutralizing VEGF-A. Subsequently, Several TKIs have been approved by the FDA and are currently an integral part of the treatment of multiple cancers (Table 1).

**Table 1:** Vascular endothelial growth factor signaling pathway inhibitors approved by FDA for cancer treatment.

Drug	Mechanism of action	FDA-approved indications
*Bevacizumab*	Monoclonal anti-VEGF antibody	mCRC, cervical, endometrial, NSCLC, GBM, mHCC, mRCC, ovarian, primary peritoneal and fallopian tube cancer
*Ramucirumab*	Recombinant monoclonal anti-VEGFR2 antibody	Gastric cancer, HCC, mNSCLC
*Aflibercept*	Recombinant fusion protein decoy for VEGF-A, VEGF-B and PIGF	mCRC
*Sunitinib*	Multi-TKI inhibitor (VEGFR, PDGFR, FLT3, CSF-1R, RET)	GIST, RCC, pancreatic neuroendocrine tumors,
*Sorafenib*	Multi-TKI inhibitor (VEGFR, PDGFR, RAF, KIT, FLT3, RET)	HCC, RCC and thyroid carcinoma
*Axitinib*	Multi-TKI inhibitor (VEGFR, PDGFR, c-KIT)	RCC
*Pazopanib*	Multi-TKI inhibitor (VEGFR, PDGFR, FGFR, c-KIT, RAF, c-KIT, Lck, c-Fms)	RCC, soft tissue sarcoma
*Regorafenib*	Multi-TKI inhibitor (VEGFR, PDGFR, RET, FGFR, TIE2, RAF-1, BRAF)	GIST, mCRC, HCC
*Vandetanib*	Multi-TKI inhibitor (VEGFR, EGFR, RET, BRK, TIE2)	Medullary thyroid cancer
*Cabozantinib*	Multi-TKI inhibitor (VEGFR, RET, AXL, KIT, MET, TIE2)	HCC, RCC, thyroid cancer (medullary or undifferentiated)
*Lenvatinib*	Multi-TKI inhibitor (VEGFR, PDGFR, FGFR, KIT, RET)	Thyroid cancer, RCC, HCC and endometrial carcinoma
*Tivozanib*	Multi-TKI inhibitor (VEGFR, PDGFR, c-KIT)	RCC
*Ponatinib*	Multi-TKI inhibitor (BCR-ABL, T315I, VEGFR, PDGFR)	CML, ALL (T315I + Philadelphia +)

VEGF, Vascular endothelial growth factor; VEGFR, vascular endothelial growth factor receptor; PIGF, placental growth factor; PDGFR, platelet derived growth factor receptor; mCRC, metastatic colorectal cancer; mNSCLC, metastatic non-small cell lung cancer; GBM, glioblastoma multiforme; mHCC, metastatic hepatocellular carcinoma; mRCC, metastatic renal cell carcinoma; GIST, gastrointestinal stromal tumor; CML, chronic myeloid leukemia; ALL, acute lymphoblastic leukemia.

Hypertension (HTN) is the most common vascular toxicity associated with VSP inhibitors. Approximately 25% of patients initiated on bevacizumab and sunitinib (a multi-target VEGF signaling pathway inhibitor) will develop HTN [7], [8]. Clinical trials have consistently shown an increased risk of HTN with the use of VSP inhibitors. However, the exact incidence varies widely depending on the agent used, the definition of HTN, and the type of blood pressure monitoring [9]. The mechanism by which VSP inhibition cause HTN is still not fully understood. However, studies have found VSP inhibition to reduce NO pathway metabolites and NO-dependent processes leading to endothelial dysfunction and impairment of vasodilation. In a study of patients on the TKI regorafenib, levels of oxidation products of NO were reduced while patients were on the drug but recovered while they were off therapy. These findings suggest that the effects of VSP inhibition on vascular, and renal function are temporary and resolve once VSP inhibitors are stopped. Another study of patients with renal cell carcinoma receiving TKIs and bevacizumab demonstrated reduction in levels of the downstream messenger of NO cyclic guanosine monophosphate (cGMP) in urine [10], [11]. Additionally, inhibition of the VSP affects endothelial cell survival, leading to a phenomenon known as capillary rarefaction and consequently an increase in systemic vascular resistance. This was highlighted in a series of patients with metastatic colon cancer who received bevacizumab and developed anatomic and functional decreases in skin capillary density and blood flow after six months, with patients receiving higher cumulative doses showing greater capillary rarefaction [12], [13] (Fig. 1).

**Fig. 1:**
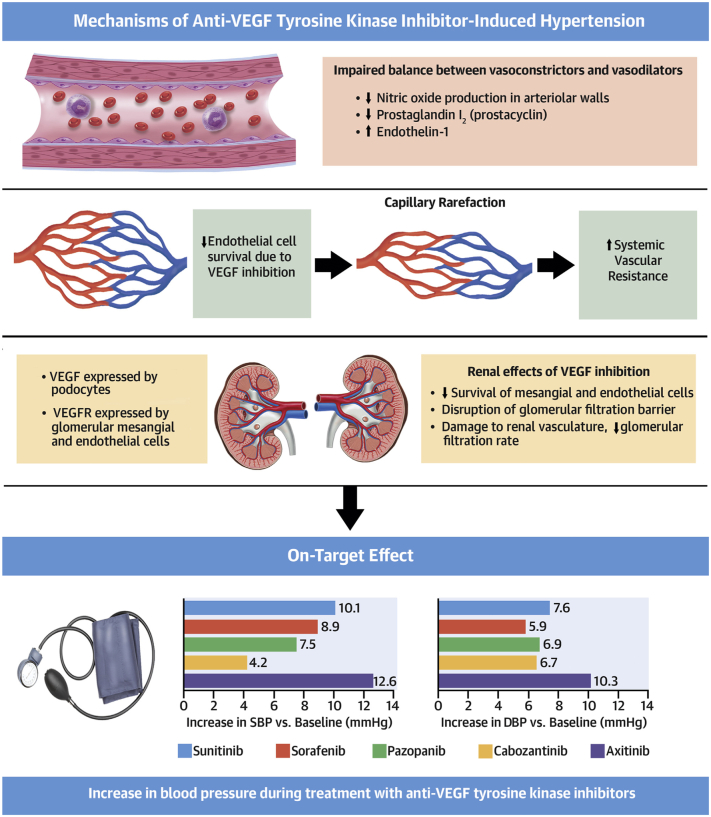
Hypertension Induced by Anti-Vascular Endothelial Growth Factor Tyrosine Kinase Inhibitors: Mechanisms and Outcomes. Anti-vascular endothelial growth factor tyrosine kinase inhibitors result in increased vasoconstrictor levels, decreased vasodilator levels, vascular rarefaction, and renal damage, which are possible mechanisms behind antiangiogenic-induced hypertension. *Reproduced with permission from Waliany* et al. *JACC CardioOncol*. *2019*;*1*(*1*):*24–36*.

In a cohort of 1120 patients with different malignancies treated with anti-VEGF therapies, Hamnvik et al. analyzed the risk of developing HTN associated with different baseline characteristics. In multivariate analysis, HTN (odds ratio [OR], 1.56; 95% confidence interval [CI], 1.27–1.92), age >60 years (OR, 1.26; 95% CI, 1.06–1.52), and obesity (OR, 1.26; 95% CI, 1.04–1.53) were identified as independent predictors of HTN induced by VSP inhibition [14]. There are no specific guidelines for the treatment of HTN secondary to VSP inhibition. However, careful baseline blood pressure measurements, detailed evaluation of cardiovascular risk factors, and treatment of pre-existing HTN and comorbidities are recommended. Treatment of VSP inhibition-induced HTN remains largely dependent on existing comorbidities and baseline characteristics, with current guidelines recommending the use of specific antihypertensive agents in patients with certain conditions such as heart failure, ischemic heart disease, chronic kidney disease, Diabetes mellitus, cerebrovascular disease etc. [15] Nonetheless, in patients with HTN primarily driven by VSP inhibition, data suggest that calcium channel blockers, angiotensin-converting enzyme inhibitors, and potassium-sparing diuretics are effective and should be considered initial therapies [13]. There is limited data on the potential value of other antihypertensive agents such as nitrates in this particular population. Importantly, non-dihydropyridine calcium channel blockers should be avoided as they are CYP3A4 inhibitors and could potentially interact with the metabolism of some VSP inhibitors such as sunitinib and sorafenib [16]. The Cardiovascular Toxicities Panel, convened by the Angiogenesis Task Force of the National Cancer Institute Investigational Drug Steering Committee, recommends that blood pressures be monitored weekly during the first cycle of VSP inhibitor therapy, and once stable blood pressures are achieved, depending on the level of risk for complications, the evaluation schedule might be more aligned with routine clinical evaluations or home measurements every 2–3 weeks until treatment is stopped. The same panel recommends a target of less than 140/90 mmHg for blood pressure control in patient receiving VSP inhibitors and less than 130/80 mmHg for patients with diabetes and/or chronic kidney disease [17].

As VSP inhibitors are increasingly used, additional studies are needed to better understand several aspects of HTN induced by VSP inhibition, such as the exact mechanisms by which VEGF signaling leads to vascular dysfunction, the optimal approach to blood pressure monitoring, and which antihypertensive agents and blood pressure targets could lead to improved outcomes. Answers to these questions would minimize unwanted effects and improve patient outcomes.

## Funding

This research received no specific grant from any funding agency in the public, commercial, or not-for-profit sectors.

## Declaration of competing interest

The authors declare that they have no known competing financial interests or personal relationships that could have appeared to influence the work reported in this paper.
